# Primary Neuroendocrine Breast Carcinoma in a 13-Year-Old Girl: Ultrasonography and Pathology Findings

**DOI:** 10.1155/2017/7915806

**Published:** 2017-09-10

**Authors:** Mazamaesso Tchaou, Tchin Darré, Koué Folligan, Akomola Sabi, Lantam Sonhaye, Azanledji Boumé, Akila Bassowa, Solange Adani-Ifé, Gado Napo-Koura

**Affiliations:** ^1^Department of Radiology, The University Teaching Hospital of Lomé, Lomé, Togo; ^2^Department of Pathology, The University Teaching Hospital of Lomé, Lomé, Togo; ^3^Department of Histology-Embryology, The University Teaching Hospital of Lomé, Lomé, Togo; ^4^Department of Endocrinology and Nephrology, The University Teaching Hospital of Lomé, Lomé, Togo; ^5^Department of Pediatric Surgery, The University Teaching Hospital of Lomé, Lomé, Togo; ^6^Department of Obstetrics and Gynecology, The University Teaching Hospital of Lomé, Lomé, Togo; ^7^Department of Clinical Oncology, The University Teaching Hospital of Lomé, Lomé, Togo

## Abstract

Neuroendocrine carcinoma (NEC) of the breast is a rare disease and has been scarcely reported by African authors. The authors report a case of breast NEC in a 13-year-old African girl initially diagnosed as an atypical adenofibroma by ultrasonography. Ultrasound-guided biopsy and conventional histological examination indicated two potential diagnoses: primary malignant non-Hodgkin's lymphoma and undifferentiated carcinoma. According to immunohistochemistry performed on paraffin blocks in France, infiltrating ductal carcinoma with a strong neuroendocrine component was confirmed by CD56, CD57, and chromogranin A markers.

## 1. Introduction

Neuroendocrine carcinomas (NECs) constitute a very rare entity, affecting mainly the bronchopulmonary system and gastrointestinal tract [1, 2]. Breast localizations are unusual and represent less than 0.1% of mammary cancers and less than 1% of neuroendocrine tumors [1]. Most publications in radiology describe nonspecific suspicious findings and do not indicate consistent imaging characteristics of this particular carcinoma by all the available modalities, including ultrasonography, mammography, and MRI [3]. In pathology, diagnosis is based on morphological criteria and confirmed by the expression of neuroendocrine markers (chromogranin and synaptophysin) in more than 50% of tumor cells [4]. There are two forms of breast NEC: the pure form exclusively composed of neuroendocrine cells and the mixed or composite form that is less well-differentiated [2, 4]. The composite form often poses diagnostic difficulties, especially for laboratories lacking immunohistochemical techniques, which explains the extreme rarity of the cases reported by African authors [5]. We report a primary composite neuroendocrine carcinoma in a 13-year-old Togolese girl confirmed by immunohistochemistry. We detail the epidemiological, morphological, and immunohistochemical aspects of this rare tumor.

## 2. Case Report

**Figure 1:**
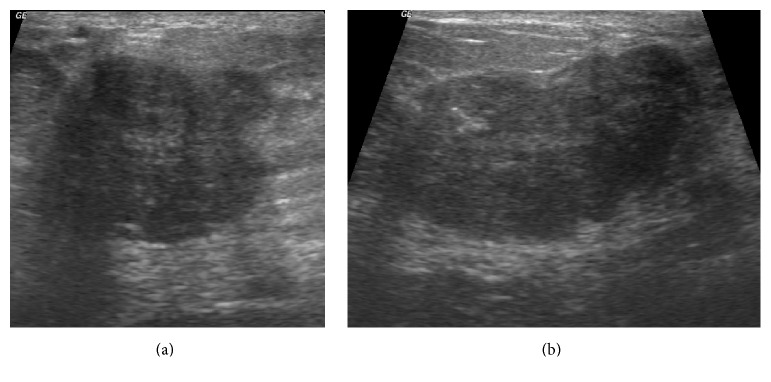
Transverse (a) and longitudinal (b) ultrasonography images showing a solid hypoechoic and heterogeneous mass with microlobulated contours, measuring 3.6 cm × 2.9 cm × 2.3 cm, with long transversal axis of the external superior quadrant of the right breast (collection of the Department of Imaging, the University Teaching Hospital of Lomé).

**Figure 2:**
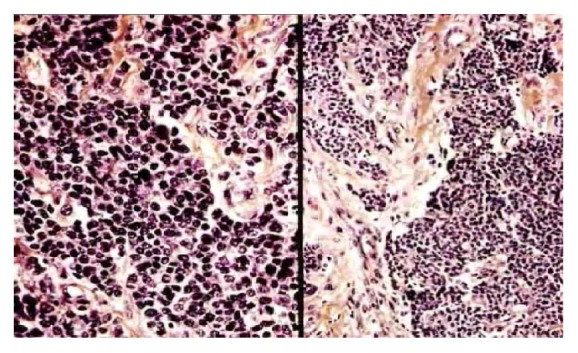
Neuroendocrine carcinoma of the breast (HES; ×100): tumoral diffuse proliferation due to medium and large cells invading the gland and oppressing lobules and ducts (collection of the Pathological Anatomy Laboratory, the University Teaching Hospital of Lomé).

**Figure 3:**
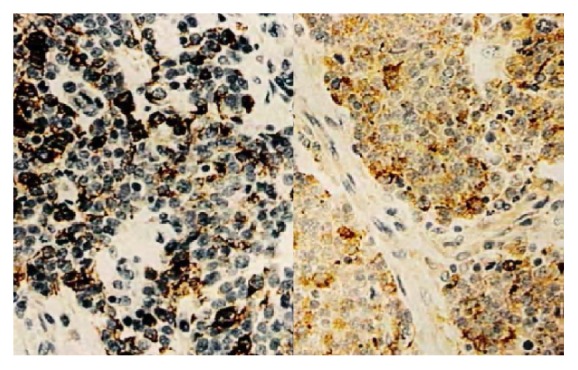
Neuroendocrine carcinoma of the breast (IHC; ×100): chromogranin A test positive for neuroendocrine tumor cells and negative for leukocyte markers (collection of the Pathological Anatomy Laboratory, the University Teaching Hospital of Lomé).

A 13-year-old girl with no remarkable past medical history and no family history of breast cancer presented with a palpable mass in her right breast, which had been evolving for 7 months. On physical examination, an approximately 4 cm firm and mobile nodule was identified. There were no axillary nodes, skin abnormality, nipple retraction, or abnormal nipple discharge. The nodule was located in the superior-external quadrant of the right breast. Due to her young age, the patient underwent only ultrasonography; no mammography was performed. Ultrasonography showed an oval hypoechoic, heterogeneous mass with microlobulated contours, measuring 3.6 cm × 2.9 cm × 2.3 cm, with long transverse axis (Figure 1). The mass was considered as atypical adenofibroma and categorized as ultrasound BI-RADS 3. On the demand of the parents, an ultrasound-guided biopsy with a 16G automatic core needle was performed before surgical ablation. On conventional histological examination, a diffuse tumor proliferation, made up of small round cells with hyperchromatic nucleus and a scant limited amphophilic cytoplasm, massively infiltrating the gland and penetrating the lobules and channels (Figure 2) was observed. This aspect had evoked two diagnoses: primary malignant non-Hodgkin's lymphoma and undifferentiated carcinoma. The paraffin blocks were sent to France for immunohistochemical analysis. Immunohistochemical studies demonstrated infiltrating ductal adenocarcinoma expressing cytokeratin and membrane epithelial antigen associated with a neuroendocrine population expressing CD56, CD57, and chromogranin A. The leukocyte markers were negative (Figure 3). TTF1 and CDX2 markers were not expressed by the tumor cells. Therefore, the case was a composite form of breast NEC. Abdominal ultrasound and thoracoabdominal CT scan performed excluded any secondary site or any other nonmammary primitive site. The treatment consisted of tumor surgery followed by chemotherapy. The patient died 5 months after diagnosis, owing to local recurrence and metastasis.

## 3. Discussion

NECs are very rare tumors, with an incidence of approximately 0.7 cases per 100,000 inhabitants, commonly located within the digestive tract. Mammary localization is very rare, accounting for less than 0.1% of all breast cancers and less than 1% of neuroendocrine tumors [1, 5]. Our observation illustrates the diagnostic difficulties encountered in practice by underequipped pathological laboratories in Black Africa (e.g., absence of immunodetection technology and electron microscopy). Indeed, in our case, if the paraffin blocks were not sent to France, the diagnosis of NEC would be impossible to affirm. The lack of adequate technical equipment and advanced technologies in pathology laboratories noticed in the majority of African countries can explain a large part of the extreme rarity of the cases of NEC of the breast reported by African authors [6]. We reported a case of neuroendocrine carcinoma of the right breast in a young Togolese girl with fatal evolution. NEC occurrence at this age is rarely described in the literature, it is common in elderly patients, specifically between the sixth and seventh decades [2, 4, 6].

In ultrasonography, it is common to misdiagnose breast masses, considering them as benign or probably benign lesions, or adenofibroma [7]. In these situations, fine needle aspiration biopsy or ultrasound-guided core needle biopsy is necessary [3, 7]. In their recent literature review, Collado-Mesa et al. [3] noticed that imaging features of primary neuroendocrine tumor of the breast have been previously described by only a small number of case reports [8–13]. The published cases describe nonspecific suspicious findings and do not indicate consistent imaging characteristics of this particular carcinoma. On ultrasonography, which is the only breast imaging technique performed in this case, lots of aspects have been described. The typical appearance of this cancer has been reported as a hypoechoic or heterogeneous mass, with irregular shape or microlobulated margins and with normal sound transmission [10, 14]. In some situations, especially in older women, mammography can be crucial for the final diagnosis by revealing a distinctive mass with microcalcifications [15]. Imaging such as ultrasound, CT scan, and even PET scan if available can help in excluding another primary site of neuroendocrine tumor [15].

Histologically, the breast NEC is characterized by cell proliferation appearing as pseudorosettes invading the surrounding adipose tissue, with a richly vascularized small or fibrous stroma and small monomorphic cell elements with rather irregular nuclei and weak mitotic activity [1, 16]. Depending on the cell type, grade, degree of differentiation, and presence of mucin production, several subtypes are defined in the WHO classification. Solid NEC, small cell carcinoma, and large cell NEC [6] have been noted. The pure form expresses a high degree of histological differentiation, whereas the composite form, apart from the neuroendocrine expression, presents either a sarcomatous component or an epithelial component [6, 17]. Our case included a composite form of the tumor, and the histological description suggested that an undifferentiated epithelial tumor initially evoked non-Hodgkin's lymphoma. In the absence of immunodetection techniques, it is difficult to confirm the diagnosis of breast NEC, especially in its mixed form, using conventional histology [17]. Even though immunohistochemical techniques have made significant progress in the diagnostic accuracy of the majority of tumors [18, 19], they are still inaccessible for the majority of African countries. The immunohistochemical study in our case presented several diagnostic advantages, including the positivity of the CD56, CD57, and chromogranin markers, for the diagnosis of NEC. Moreover, the immunopositivity of cytokeratin and epithelial membrane antigen indicated the epithelial component of our case, suggesting a composite or mixed form. Lastly, negative result for lymphocytic leukocyte markers made it possible to rule out non-Hodgkin's lymphoma, which was initially evoked. In practice, the distinction between the two forms of breast NEC is necessary because their prognosis is significantly different [20, 21]. Primary neuroendocrine cancer of the breast must be distinguished from a metastatic lesion from other sites. Some markers such as TTF1 even if it is positive in only 55% of primary lung neuroendocrine tumors help to exclude lung primary NEC [15]. Positive nuclear CDX2 expression confirmed intestinal derivation [21]. Thoracic and abdominal imaging screening is helpful.

The treatment of endocrine tumors of the breast mainly comprises surgical tumor removal. The indications of chemotherapy and radiotherapy are the same as for other breast cancers. The indications of hormone therapy and immunotherapy are not coded because their effects remain uncertain [22]. In our case, the patient benefited only from surgery and chemotherapy.

## 4. Conclusion

Breast NEC is very rare and has poor prognosis. Further, its occurrence in a young patient is unusual. As there are no imaging specific features, the diagnosis of certainty is based on immunohistochemical analysis, which makes it possible to differentiate the pure forms from the composite forms. This case of a composite breast NEC also illustrates the diagnostic difficulties encountered by underequipped pathology laboratories in developing countries, explaining, in part, the extreme rarity of cases reported by African authors.

## Additional Points

*Availability of Data and Materials.* All data generated or analysed during this study are included in this published article.
